# Facilitating Personal Recovery in the Community: a Qualitative Evaluation of a Peer Support Group for Adults with Severe Mental Health Challenges in Rural Australia

**DOI:** 10.1007/s10597-026-01600-1

**Published:** 2026-03-12

**Authors:** Anton Isaacs, Anna Baker

**Affiliations:** 1 School of Rural Health, Monash University, Melbourne, Australia; 2 Mental health services, Latrobe Regional Hospital, Traralgon, Australia

**Keywords:** Severe mental illness, Mental Health Recovery, Needs Assessment, Health Services Needs and Demand, Activities of Daily Living, Social Work, Psychiatric, Psychiatric Rehabilitation, Health Services Research, Health Priorities, Mental Health Services, Peer Support Groups

## Abstract

Individuals with severe mental health challenges [SMHCs] often have unmet needs that are beyond the scope of traditional mental health services. These needs are typically met by community agencies, charities and peer support groups. Happy Chat [HC] is one such peer-support group whose goal is to address social isolation, and loneliness by creating a safe space to facilitate the development of informal friendships among individuals with SMHCs in the community. Nineteen participants were interviewed as part of a qualitative evaluation of HC. Data were subjected to thematic analysis. Four categories emerged from the data. They were: Difficulties faced by persons with SMHCs (6 codes), Supports provided by HC (15 codes), Outcomes achieved (15 codes) and Continuing challenges (4 codes). Key supports reported by participants included a welcoming and non-judgemental space, that is inclusive and safe, continuing human connections, ongoing social, emotional and general support, as well as support and care during a crisis. Key outcomes reported by participants included improved confidence and motivation to leave the house, having a place of comfort and a sense of belonging, improved self-care, and social life, a new identity, and a renewed sense of hope and purpose. Four supports and one outcome were identified as being more closely aligned to the CHIME framework of personal recovery. Ongoing challenges reported by participants such as limited funding, are common in small, resource-limited peer support groups. Further research is needed to demonstrate any association between meeting basic and psychosocial needs and achieving personal recovery among individuals with SMHCs.

**Supplementary Information:**

The online version contains supplementary material available at 10.1007/s10597-026-01600-1.

## Background

Individuals experiencing severe mental illness and neurodiversity are known to experience challenges such as social isolation, homelessness, stigma and discrimination (Han et al., 2022; Jenkins et al., 2023; Kerman & Stergiopoulos, 2024; Pellicano et al., 2022). International evidence suggests that mental health services are also fraught with difficulties. They are often not fit for purpose, are fragmented and operate in isolation (Melamed et al., 2019; Nicaise et al., 2014; Petrie et al., 2021; Royal Commission into Victoria’s Mental Health System, 2021; Triliva et al., 2020). As a result, their clients often disengage with the mental health system altogether (Isaacs et al., 2017). The term, severe mental health challenges [SMHC] has been used in this paper to refer to individuals who have been diagnosed with a mental health condition that has severely impacted their ability to carry out activities of daily life.

The primary objective of contemporary mental health services is to facilitate personal recovery among their clients (Commonwealth of Australia, 2013). Personal recovery for individuals with SMHCs extends beyond mere symptom relief and is founded upon the principles of connectedness, hope and optimism, identity, meaning and purpose, and empowerment, often referred to as the CHIME framework (Leamy et al., 2011). When individuals are diagnosed with a severe Mental Illness [SMI], they often get disconnected from family and friends and lose hope, meaning and purpose. They also experience a loss of choice and control in their lives and begin to recognise a new version of themselves that is different from what it was prior to their illness (Isaacs et al., 2022). Such individuals are considered to be progressing towards personal recovery when they experience a sense of connection with family, friends, and society, maintain hope for the future, cultivate an identity distinct from their illness, and feel empowered through engagement in employment and volunteer activities (Leamy et al., 2011). Moreover, individuals with SMHCs can attain personal recovery even in the absence of complete symptom remission (Anthony, 1993). They achieve this by establishing meaningful roles, relationships, and activities that impart purpose to their lives, despite the persistent challenges associated with their illness.

It is also well known that people who are exposed to unfavourable socioeconomic circumstances are more vulnerable to poor mental health (Kirkbride et al., 2024). Individuals with SMHCs have reported unmet socioeconomic needs such as those related to daytime activities, someone to spend time with, accommodation, food, and transport (Isaacs et al., 2019; Thornton, 1977; Whiteford et al., 2017). The ongoing state of unmet needs in this group of service users causes them substantial psychological distress (Riley. M & Moses, 1977).

Personal recovery-oriented services therefore need to also take action on social inclusion and social determinants (Commonwealth of Australia, 2013). This is in contrast to public mental health services which typically follow the biomedical model and are oriented towards reducing symptoms and improving functioning (Deacon, 2013; Lebowitz & Appelbaum, 2019). Such services are not designed to provide holistic care or social determinant-informed interventions (Claisse et al., 2024). Indeed, the literature suggests that medication and psychotherapy continue to have limited success in improving long-term recovery outcomes in persons with SMHCs (Jääskeläinen et al., 2013; McBain et al., 2020).

Individuals with SMHCs who reside in rural locations also experience challenges such as a lack of services (Merwin et al., 2003) which is worsened when they are geographically isolated (Shiner et al., 2022). In addition, stereotypical views of mental illness are known to be common in rural areas (Boyd & Parr, 2008), where mental illness is often associated with incompetence and dangerousness (Larson et al., 2012). As a result, social stigma towards mental illness is pervasive in rural areas. While the levels of stigma vary between communities, smaller communities tend to hold more stigmatising attitudes (Larson et al., 2012). The fear and social stigma associated with mental illness in rural communities can result in people with SMHCs being ostracised and excluded (Sutton & Isaacs, 2021).

Community-based mental health agencies such as recovery centres (Whitley et al., 2012), community-based peer recovery services (Kowalski, 2020) and charities (Claisse et al., 2024) are stepping in to provide the type of care needed for these individuals, to better support their recovery and overall wellbeing. These agencies provide opportunities for people to come together locally and be involved in activities that include social interaction with peers in a safe space (Claisse et al., 2024). Such interventions have been referred to as personal recovery facilitators that are primarily aimed at supporting personal recovery processes (Isaacs et al., 2022). Services provided by these agencies have been found to be valuable when undertaken in conjunction with existing community healthcare services which have a clinical focus (VanHorssen et al., 2024) and need to be ongoing in order to ensure long-term recovery.

Peer support has been recognised as filling a gap in mental health care for persons experiencing mental illness in rural and remote communities in Australia (Jackson et al., 2021). Peer workers are known to be a source of hope for individuals and communities experiencing adversity. They adopt a strengths-based approach to care although the mechanisms by which they achieve positive outcomes are not fully understood (Vayshenker et al., 2016). It has also been reported that people with SMHCs who are involved in peer support initiatives, have higher levels of community integration (Repper & Carter, 2011). Characteristics of peer work include not being an expert over the person being supported, but rather encouraging them to find their own ways forward. This is achieved by gently championing them to find their voice and advocate for themselves, as well as by connecting them to family, friends and community and to the services they need (Jackson et al., 2021). By helping individuals recognise their own abilities and prioritising relationships, rural peer workers contribute to improved functioning, better physical and mental wellbeing, community involvement and accommodation among people with SMHCs (Dunstan & Anderson, 2018).

However, community-based mental health agencies often face obstacles in sustaining their programs (Larrieta et al., 2023). Budgetary constraints that prevent organisations from continuing the important work they do, are foremost among them (Claisse et al., 2024). One such organisation is Happy Chat based in the small rural town of Stanthorpe, Queensland.

### Happy Chat

Happy Chat [HC] is an independent, rural, self-help, peer-support group that is run by volunteers with a lived experience of SMHCs who are in various stages of recovery. Their primary mission is to address social isolation, and loneliness by creating a safe space to assist the development of informal friendships. In doing so, they facilitate meaningful connections, support individuals entering formal services (if desired) and address ad hoc problems before a crisis develops. HC runs activities such as weekly peer-support group meetings, ‘dungeons and dragons’ group, and a monthly barbecue at the community garden. They also provide financial support, as well as assistance before, during and after hospital admission.

HC has 60 participants who are aged between 15 and 75 + years. On average, about 20 of them attend activities every week. Most participants have a psychiatric diagnosis, are at high risk of social isolation and are socio-economically disadvantaged. For many, HC is their only opportunity to connect, exchange information and socialise with other people. Only about 25% of HC participants have a current NDIS [National Disability Insurance Scheme] package. The NDIS provides funding for supports and services for people with disabilities (NDIS, 2024). Anecdotally, HC appears to be making a substantial difference to the lives of their volunteers and participants, although a formal evaluation of its activities has not been undertaken. Moreover, peer support groups for people with SMHCs in rural Australia have received limited empirical attention and this work begins to fill that gap. The aim of this evaluation is to obtain participant views of how, if ever, HC has helped persons with SMHCs. This study also explored whether the support provided by HC facilitated personal recovery in its members.

## Methodology

### Study Design

This qualitative evaluation had two parts. The first sought to explore participant views on the ways in which they were supported by HC and the difference that support made to their lives. This part was informed by Qualitative Description [QD] (Sandelowski, 2000) the goal of which is to provide a straight description of an experience in easy to understand language without much interpretation (Sullivan-Bolyai et al., 2005). QD was chosen as the preferred method of analysis because lived experience accounts of individuals with SMHCs that are presented as is, are easily understood by service providers and policy makers. As a result, this method is commonly utilised in health services research (Sandelowski, 2000; Sullivan-Bolyai et al., 2005).

The second part of the evaluation explored if the supports provided by HC and the outcomes reported by participants aligned with the domains of the CHIME framework of personal recovery (Leamy et al., 2011). Previous research has suggested that the different needs of individuals with SMHCs as listed in the Camberwell Assessment of Need (Phelan et al., 1995) could be related to the domains of the CHIME framework (Isaacs et al., 2020). For instance, when needs related to accommodation, food, benefits, psychological distress and finances were met, it could result in improving hope and optimism. Similarly, connectedness can be facilitated when needs related to company, service access, transport, etc., were met (Isaacs et al., 2020). The study adopted the COREQ guidelines for qualitative research (Tong et al., 2007). See supplementary files for COREQ checklist.

### Setting

HC is based in Stanthorpe, a rural town in South-eastern Queensland with a population of about 5200. Indicators of social and economic status for this town show higher levels of disadvantage when compared that of the state and country. (Australian Bureau of Statistics, 2021). For instance, participation in the labour force of those aged 15 years and older was 47% for Stanthorpe. Corresponding state and national figures were 61.6% and 61.1% respectively. Similarly, median household weekly income for Stanthorpe was $970 when compared to $1675 and $1746 for the state and country respectively (Australian Bureau of Statistics, 2021).

### Reflexivity

The lead author of this study is a senior researcher with expertise in mental health services research, and in research involving persons with SMHCs. The second author is a Lived Experience Worker (Byrne et al., 2021) in a regional mental health service and has previously co-authored similar research. Both authors are strong advocates of the importance of lived experience in designing and implementing models of care and services for people with mental health challenges. The lead author was invited by HC to undertake the evaluation.

### Participant Recruitment

Recruitment of participants was undertaken face-to-face (hard copy invitations, group information sessions over three consecutive HC meetings), as well as by email, Messenger and Discord in April 2025. Some Happy Chatters requested for their support person/carer to also be included. A total of 26 Happy Chatters and their carers were invited to participate in the study. Those who chose to participate in the evaluation returned signed consent forms. The lead author then contacted them to organise a suitable time for the interview.

### Data Collection

Semi-structured interviews were conducted with participants of HC either by videoconference (ZOOM) or telephone. As advised by the Human Research Ethics Committee, participants were asked the following questions by the lead researcher (interviewer) prior to the interview to assess their capacity to participate in the research:

1. What condition/s have you been diagnosed with?
2. What challenges have you faced due to your condition/s?
3. Is there any specific reason why you consented to participate?
4. How are you feeling at the time of the interview?
5. Do you feel capable and comfortable to participate?

Participants were deemed capable of participating in the research if they were able to appreciate their situation realistically, understand their condition and its consequences, provide a logical reason for participation and communicate the same in a clear and logical manner. Once participants were found to be capable of participating in the research, they were asked the following questions.

1. Before you joined HC, what, if any, specific challenges or needs were you experiencing?
2. In what ways has HC helped you address or overcome these challenges or needs?
3. How, if ever, has life changed for you after joining Happy Chat?
4. In your opinion, how does HC help people such as yourself?
5. Are there any continuing challenges that you experience?

Interview questions were jointly developed by the researchers and the HC coordinator. A distress protocol adapted from Whitney and Evered (2022) was established and approved by the human research ethics committee prior to the study. Interviews lasted between 20 and 45 min, were audio recorded and professionally transcribed. Transcripts were coded using an alpha-numeric code (e.g. 4 M stands for Participant 4, Male). Participants were reimbursed for their time with an honorarium of $40 provided in the form of supermarket vouchers.

### Data Analysis

Data were initially analysed inductively. Chunks of data that captured a rich qualitative aspect of a phenomenon were assigned codes. Initial coding was done by the lead author. The second author then independently reviewed the codes and made necessary changes. Codes were finalised following agreement by both authors by combining, separating or renaming them to ensure that they accurately reflected the data they represented. Codes were then grouped together by the first author, to form tentative categories which were finalised following review and revisions by both authors. Creswell and Miller (2000) suggest that respondent validation (member checking) is better conducted with ‘interpreted pieces such as themes and patterns emerging from the data rather than the actual transcripts’. Accordingly, a copy of codes, categories and representative quotes was sent to all participants for comments. Upon finalizing the codes and categories, each code from the categories of Supports provided by HC and Outcomes Achieved was matched with the five domains of the CHIME framework by two consenting study participants who performed the task collaboratively. This matching exercise was also conducted independently by the lived experience researcher of this study. Supports and outcomes were deemed to be more closely aligned with the CHIME framework when both participants and the lived experience researcher matched them with three or more CHIME domains.

## Results

Nineteen individuals participated in the study, of which 4 were carers. Of those who experienced SMHCs, 8 (53%) were in the 50–59-year age group, 7 (47%) had school-level education, and 8 (53%) lived in their own home. Of those who experienced SMHCs, all had a mental illness for a period of 10 years or more, 11 (58%) participants were hospitalised for their illness one or more times and fourteen (93%) had been with HC for more than one year. See Table 1. Four categories emerged from the data. They were: Difficulties faced by persons with SMHCs (6 codes), Support provided by Happy Chat (15 codes), Outcomes achieved (15 codes) and Continuing challenges (4 codes). Categories, and their codes are given in Table 2. The full list of codes and their representative quotes are given in Supplementary Tables. Categories and codes with key representative quotes are briefly discussed in the text below.

**Table 1:** Demographic characteristics of participants (*N* = 15 participants with SMHCs + 4 carers)

Characteristic		No.	Characteristic		No.
Age	20–29	1	Diagnosis* (reported by participants)	Schizophrenia	5
	30–39	1		Obsessive compulsive disorder	5
	40–49	4		Bipolar disorder	5
	50–59	8		Depression	5
	60–69	1		Generalised anxiety disorder	6
Sex	M	8		Chronic post-traumatic stress disorder	7
	F	6		Autism spectrum disorder	3
	Non-binary	1		Attention-deficit hyperactivity disorder	5
Highest Education	Junior school	1		Panic disorder	1
	High school	6		Eating disorder	1
	Certificate	2			
	Diploma	3	Illness duration	10–19 yrs	5
	Graduate	2		20–29 yrs	3
	Postgraduate	1		30–39 yrs	4
Occupation	Disability Pension	7		40–49 yrs	2
	Age pension	2		50 + yrs	1
	Job seeker	3	Hospitalisations	0	4
	Self-employed	2		1–5	7
	Support worker	1		6–10	2
Housing	Own house	8		10+	2
	Living with family	1	Time with HC	6months – 11months	1
	Renting	3		1 year – 4 years	7
	Public/social housing	2		5 years – 9 years	4
	Homeless	1		10 years +	3
Has a carer	Yes	6	**Carer participants**		4
	No	9			

*Participants reported having multiple diagnoses. Each diagnosis reported by participants has been counted

**Table 2:** Categories and codes (Code tree) generated from the qualitative evaluation of Happy Chat, a peer support group for persons with severe mental health challenges

1. Difficulties faced by persons with MHCs	3. Outcomes achieved
Isolation	Motivation to leave the house
Homelessness	Improved confidence
Discrimination	Enabled a sense of belonging
Disrespect	A place of comfort
Loss (of identity, self and relationships)	Safe to be me
Worsening of mental illness	Improved health and wellbeing
2. Supports provided by Happy Chat	Improved self-care
Welcoming and Non-judgemental	Improved social life and social skills
Sharing a meal and a chat	A new identity
Inclusive	A new sense of purpose
Safe space	A new sense of hope
Human connections	Employment
Building hope through kindness	Realisation that it is the system that has failed and not us
Social and emotional support	4. Continuing challenges
General support	Dependence on one person
Helping each other	Lack of funds
Enabling agency	Managing difficult behaviours
Monitoring wellbeing	Continuing discrimination in society
Crisis care and support	
Advocacy	
Continuing connections	
Continuing support	

### Difficulties Faced by Persons with SMHCs

As the label suggests, this category relates to the difficulties faced by participants prior to joining HC. Isolation was a major challenge faced by participants. Isolation occurred as a result of community stigma, a fear of rejection, a lack of support services or homelessness. For instance, one participant said:

… you feel isolated from the rest of the community because you don’t know anyone [who] has the same illness and when you talk to them about it, they look at you like you’re crazy or you’re gonna stab them or something like that, you know? – 1 M.

Similarly, another said:

I went out, not on my own at all for that period of time. And, just that incredible fear of rejection and confrontation and all that kind of stuff. – 10 F.

Homelessness was also considered a major problem among participants in the area with some resorting to ‘couch surfing’ or living in their cars or shipping containers.

There is a fair degree of homelessness or couch surfing. Well, it’s very cold where we are. Like, this morning, I think it was six degrees. So, the winter weather is a huge problem for all of us and particularly if you’re sleeping in your car or whatever. Some of them… they sleep in the shipping containers with nothing in them, you know. So that’s a huge thing. – 10 F.

Discrimination against those with SMHCs was another challenge as stated by one participant:

If you have mental health problems in this town, you’re a second-class citizen… they treat you like garbage anyway, the hospital. – 3 M.

Participants also alluded to being disrespected, stating that they did not want to be told how to live their lives ‘by people in business suits’. As their illness developed and manifested, participants began to feel a sense of loss. This loss related to their sense of self and identity as this participant stated:

I, with the diagnosis and the unravelling of my life prior to that, I suddenly did not know who I was, anymore. Yeah. What do I like to do when my brain’s actually functioning even on an everyday level? What do I wanna do? Like, I know, I can’t sit at home forever. – 10 F.

This stigma continued to erode their sense of self and strain friendships.

I got a diagnosis, but, other than the clinical, perspective with the psychiatrist and psychologist, I didn’t really know many people with a similar one, and it was hard to talk in general as friends with my other friends because they didn’t quite understand. And I lost [friends] and that was hard just because of the stigma that came with the diagnosis. – 11 F.

### Supports Provided by HC

This category relates to participant views on the different ways HC helped and supported them. HC as a group was reported to be welcoming and non-judgemental, where one could go when they wanted to and stay as long as they liked. One participant said:

I felt like I was welcomed, and I wasn’t overly challenged, as in people just let me be me … and so I found comfort there. That’s really important because, my anxiety, takes the form of agoraphobia and so whenever I’m outside of my comfort zone, I just feel like I’m going to be ill. I feel very sick. −13 M.

It was particularly helpful for those who had nowhere else to go and where they could be themselves, as one participant said:

No judgement whatsoever, you know… So it is for people who are struggling, have no place to go normally, and they’re looking for some place where they can, you know, really be themselves and share what they’re going through and have somebody understand and listen to what they are talking about. – 16 F.

The weekly meal which participants could share along with a chat was reported by some as the biggest success of HC.

The meals! That’s one of the biggest things that I reckon as a success at Happy Chat is that we have a meal together. Such a simple thing, but breaking bread together is a huge part of getting to know [each other]. So, access to food is amazing for your mental health… just to sit and eat together. Sometimes they take home leftovers. – 10 F.

Participants considered HC to be inclusive where one could go even if one did not want to talk to anyone but be left alone. They reported that their dietary requirements were also considered when meals were prepared.

Happy Chat was considered to be a safe space for participants with different types of problems. Some reported feeling ‘emotionally comfortable’ where they could talk about their feelings in a caring and confidential environment.

I think one of the most important things that stands out for me is the fact they had made that a very safe environment for all people. Different people have different issues. I think they had a sense of freedom. They could be themselves, and they weren’t being judged by people. It was a lovely environment. – 12 M.

Participants spoke of the human connections that were facilitated by HC. Being able to know the names of people outside of HC, being part of ‘a family’ and having ‘a new tribe around me’ were considered valuable by participants.

I mean, we range from 10 people to 30 people sometimes. And … I know 95% of all those people’s names. I see those people downtown. If it wasn’t [for me] going to Happy Chat, I wouldn’t have been able to make those relationships… to be able to talk outside of Happy Chat. So, it’s extended my network, if you wanna put it that way. – 5 M.

Participants indicated that being treated with kindness by others who were unaware of their problems inspired hope, highlighting HC’s contribution of social and emotional support. For instance, a participant stated ‘…just by voicing what I’m struggling with… lessens the load that I’m carrying.’ HC also provided general support. For instance, one member stated, ‘When I was legitimately too poor to put fuel in my motorcycle to get there, I was offered gift cards for the servo [fuel station].’ Another stated, ‘…they actually helped pay for therapy for me for a while…’. Still another said:

When I had a really difficult time with some domestic violence, they actually all, kind of banded together to help me with my pets and things. 17 F.

Participants of HC helped each other by sharing items and helping with functions of daily living. One participant reported that HC made her feel empowered and able to make choices for herself, even if they were simple choices such as whether to have lunch there or pack a take away. A volunteer at HC stated that they were like a ‘monitoring facility’ where participants who were observed to be struggling were gently and discretely supported. HC also supported their members when they were in a crisis such as when they had suicidal thoughts or were admitted to hospital. HC helped their members obtain services from external agencies, serving as their advocate. HC served as a forum to maintain connections between current and past members and as a continuing part of people’s lives. One participant stated:

I think its greatest value is that it’s a constant. It’s a constant in people’s lives; that every Tuesday morning, there’s something to get out of bed for. – 15 F.

The importance of being a constant in one’s life was described by another participant as:

And you don’t need that support for six weeks in a row. You need that support, theoretically over the year or over the span of [your illness]. So, you might be good for two months, but then you might have a really bad time for a month, and you need that extra support. And that’s what Happy Chat offered. – 5 M.

### Outcomes Achieved

This category covers participant views on the difference that HC made to their lives or how their lives had changed after getting involved with HC. HC provided motivation for participants to leave their house. A support worker and volunteer at HC explained this by saying:

My client doesn’t like to leave the house very often… he has social fears and phobias I guess, and definitely doesn’t like crowds… So, it is a great way and a great encouragement for him to get out of the house. – 2 F.

HC also helped build confidence in participants to go into public spaces, to exercise, to socialise, to help others and to go shopping. One participant said:

I actually can go shopping on my own now. I’ve actually started going to church without feeling paranoid. I’ve become a little bit more open. I can go into a café; I can talk to the staff … yeah, it’s made a huge difference like that… I’ve become more confident. Like I’ve started going to the gym three times a week and joining classes, talking to people there… I’m getting fit. I’ve got a lot of confidence to do things. – 19 M.

Participants considered HC to be a place where they could go and feel comfortable to be themselves. They also described having a sense of belonging and the joy it brought to them.

There is nothing nicer than seeing someone familiar in the main street and saying “hello”. The smile and the hug back is life giving. The sense of belonging to our community and being an active participant in it is priceless. It brings a sense of safety but also an opportunity to find joy in belonging to a community. – 9 F.

Participants reported having improved mental health, wellbeing and behaviours of self-care. They reported having improved social skills and as a result, a better social life, as one participant stated:

I am mostly socially awkward. I don’t mix with other people. A lot of that has to do with my autism. I’m not a social person but since I’ve been going there, I’ve become more social. I’ve got to talk to other people and drop my guard a bit there too. I’ve got trust issues too but I’ve learned to be more relaxed, be more myself around these people, which has definitely made a huge difference. – 19 M.

One participant described how a member who was quite unwell was able to secure a job after joining HC. Others described the realisation that they were not failures but rather, that it was the system that had failed them. Still others reported having a new sense of identity, of belonging, of purpose and of hope.

We first came to Happy Chat with the idea that all we are is a mental illness; that we are sick and disabled… When people discover that they are not defined by their mental illness, that their lives are richer than a diagnosis… there is enormous relief for the individual. More than that, it reminds them that they aren’t hopeless cases - that they are worthy of a good life… Many had lost hope before HC - I know I had. Not anymore! – 9 F.

### Continuing Challenges

In this category, participants describe their concerns and continuing challenges. For instance, HC as a group was coordinated by one individual who had SMHCs of her own with little external help.

That’s what I’m worried about… [The organiser] because she is under the pump. She has a lot on her plate for the community and that’s the only concern, because if she breaks, it all breaks. – 16 F.

A lack of funds was also reported as being a challenge.

Happy Chat doesn’t have any money… We’ve had donations in the past to pay for food, and she gets food from the food bank or Second Life or whatever it is called. – 2 F.

As far as I know, there’s a very, very tiny budget. It might only be 20 or 30 dollars a week to buy the milk and the tea and coffee and all that kind of stuff, and then some stuff they grow in the garden, they use. So that’s not adequate, you know. Like, there’s a lot of very low socioeconomic, situations for most people there. – 10 F.

Managing difficult behaviours from some of the members was also reported as a challenge.

Some became aggressive and they were told that they would have to be banned from coming for two weeks and I think one person in particular… unfortunately, went too far and they were told, ‘Please don’t come back.’ You’ve got a hard decision to be made… Yeah… But you’ve got to think of the welfare of the greater majority– 12 M.

Finally, participants bemoaned the continuing discrimination in society towards people with SMHCs.

### Comparing Supports and Outcomes with the CHIME Framework of Personal Recovery

Four of the supports provided (SP) and one outcome achieved (OA) were aligned more closely with the CHIME domains. They were Human connections (SP), Social and emotional support (SP), Continuing connections (SP) continuing support (SP) and Motivation to leave the house (OA). See Table 3.

**Table 3:** Comparison of Happy Chat supports and outcomes with the CHIME framework of Personal Recovery

Supports provided	Connectedness	Hope and Optimism	Identity	Meaning &purpose	Empowerment
Welcoming and Non-judgemental	√√	√	√	√	√
Sharing a meal and a chat	√√			√	√
Inclusive	√√	√			√√
Safe space	√√	√			
Human connections	√√	√	√√	√√	√√
Building hope through kindness	√	√√	√	√	√
Social and emotional support	√√	√√	√	√	√√
General support	√	√			√√
Helping each other	√	√	√	√√	√
Enabling agency		√	√√	√	√√
Monitoring wellbeing	√	√√	√	√√	√
Crisis care and support	√√	√√			√
Advocacy	√	√√	√	√	√√
Continuing connections	√√	√√	√√	√√	√√
Continuing support	√√	√√			√√
**Outcomes achieved**					
Motivation to leave the house	√	√√		√√	√√
Improved confidence	√	√√	√	√	√√
Enabled a sense of belonging	√√		√		
A place of comfort	√√	√√	√		
Safe to be me	√√	√	√	√	√√
Improved health and wellbeing	√	√√	√	√	√√
Improved self-care			√	√	√√
Improved social life and social skills	√√	√			√
A new identity	√	√	√√	√	√
A new sense of purpose		√	√√	√√	√
A new sense of hope	√	√		√	
Employment	√	√	√	√√	√√
Realisation that it is the system that has failed and not us	√	√√	√		√√

Participants used tick marks to identify the supports provided by HC that contributed to each of the domains of the CHIME framework. Two tick marks represent additional endorsement by the lived experience researcher of this study

## Discussion

This study aimed to explore views and experiences of participants who had received support from HC, a community-based peer support group for persons with SMHCs. Difficulties faced by participants in this study included experiencing isolation, and homelessness as well as an overarching sense of loss. Social isolation and loneliness (Jenkins et al., 2023; Rainer & Martion, 2012), homelessness (Kerman & Stergiopoulos, 2024) and the loss of relationships and friends (Topor et al., 2016) are consistent themes among persons with SMHCs. Participants in this study also reported that they experienced discrimination by health services with statements such as ‘second class citizen’ and ‘treated like garbage’. Previous reports have also indicated that people with SMHCs frequently encounter stigma and discrimination from health professionals (Cobigo & Stuart, 2010). Targeted training and social contact with individuals with SMHCs who are in recovery have shown to be effective in changing attitudes and behaviours of health professionals towards them (Stuart, 2016).

The supports provided by HC appear to have alleviated some of the challenges reported by participants and contributed to personal recovery in its members. For instance, HC was able to overcome isolation among its members by providing a safe, welcoming and non-judgemental space where people could meet others and socialise. Recovery-oriented interventions are known to work best in safe and non-judgemental environments (Winsper et al., 2020).

Social inclusion is an important priority for people with SMHCs. By providing human connections, HC was able to help its members develop new and meaningful relationships which are considered imperative to personal recovery (Schön et al., 2009). Individuals employ their shared history to build trust with each other over time. As this trust is built, participants feel safe to honestly disclose their challenges and struggles, which increases the likelihood of achieving personal recovery. In addition, witnessing progress in their peers and being reminded of and being celebrated for their own progress over months or years reinforces their belief in their own potential for change and growth. Being able to come out of their shell by overcoming their shyness and nervousness helped participants communicate with others in a safe space. This helped improve their social skills and make new friends at HC. They were able to then use those skills in the community, which in turn improved their social life. HC therefore facilitated belonging and connectedness, which are essential for personal recovery (Horsfall et al., 2018).

Positive social relationships support individuals during times of adversity by helping them cope with and overcome challenges. Social relationships achieve this by offering support (Andersen et al., 2021) and serving as a buffer to individuals during stressful events (Cohen & Wills, 1985). Social relationships can therefore reduce the severity of the stress and provide a sense of security. Social relationships also help individuals thrive by providing them with a group to belong to. The need to belong is a fundamental human need (Baumeister & Leary, 1995) and being part of a social network enables people to share activities and have conversations with people similar to them. Such supportive connections can gently shape how people process thoughts and feelings, thereby fostering emotional resilience (Lakey & Orehek, 2011). Individuals with a larger network size have reported higher levels of recovery irrespective of their psychiatric symptoms (Corrigan & Phelan, 2004). Enabling inclusion through social interventions is a developing field requiring further research (Eager et al., 2025).

Engaging with HC improved participants’ confidence, which in turn, helped them look after their health and wellbeing. Engaging with HC also gave participants the freedom to be themselves. It showed them that ‘their lives were richer than their diagnosis’ and this enabled them to feel hope and optimism for the future. Finally, HC helped individuals discover and redefine a new and positive identity and empowered them by increasing their independence.

The challenges reported by participants such as the dependence on one person, the lack of funds, managing difficult behaviours and continuing discrimination in society are not uncommon for small groups and charities with few resources. The more established recovery centres also face challenges such as the lack of model clarity, clear goals, and standardization of services (Whitley et al., 2012). There is also the challenge of some participants becoming dependent on the service (Whitley et al., 2012). However, this was not considered as a problem by participants in this study.

The supports provided by Happy Chat and the outcomes achieved by participants as a result of those supports that more closely aligned with the CHIME domains are examples of practical supports needed by individuals with SMHCs in the community. As discussed above, continuing human connections are integral to personal recovery. Having continuing human connections adds stability to one’s life and this stability is a pre-requisite to be able to draw on one’s strengths and move forward on one’s recovery journey (Hancock et al., 2018).

A strength of this model of care is its continuity. A major gap in care provided by mental health services generally is that they are episodic in nature (Iversen et al., 2025). Ongoing or continuing care rather than episodic care is essential for persons with SMHCs owing to the undulating nature of the symptoms associated with SMIs. (Adair et al., 2005; Puntis et al., 2015). People with SMHCs often struggle to access continuing models of care since they are few and far between. This gap adds to the global challenges in providing care and treatment for people with SMHCs. For instance, in the Adult Psychiatric Morbidity Survey in England, 13.6% of those with severe symptoms had an unmet treatment request in the past 12 months, compared with 0.6% of those with few or no mental health symptoms (Clery et al., 2025).

Social and emotional support (SP) particularly from peers is another unmet need for individuals experiencing SMHCs. This study adds to the developing evidence on the positive impact of social and emotional support on facilitating mental health recovery (Bjørlykhaug et al., 2022; Gidugu et al., 2015). This support takes the form of providing social opportunities and emotional support and by normalising relationships with others with similar experiences. People with SMHCs attain the motivation to leave the house (OA) when they have an opportunity for social engagement and enjoyment or life structures such as regular appointments (Jimenez et al., 2019). In this study, HC offered regular weekly gatherings and a meal that included social interaction with peers.

Key elements of the HC model are also included in other larger programs such as GROW Australia, a mental health support program based on lived experience (GROW mental health and wellbeing programs, 2026). The main elements of GROW programs include the establishment and maintenance of social relationships, mutual support and shared leadership, (Grow Australia, 2015) which are similar to that of HC. However, the evidence on the effectiveness of mental health peer support groups such as HC is still emerging. Better collaboration between clinical mental health services and community based peer support groups may go a long way in providing recovery-oriented care for people with SMHCs.

This study therefore demonstrates three key findings: First, individuals with SMHCs who face social and economic disadvantage continue to experience unmet needs within the community and within the traditional biomedical model of mental health care. Second, peer support groups such as HC demonstrate the capacity to address these unmet needs even when operating with limited financial resources and third, supports provided and outcomes achieved through meeting these needs allude to practical ways by which persons with SMHCs can achieve personal recovery.

Although previous reports have suggested that peer support group interventions may be effective in facilitating personal recovery (Lyons et al., 2021; Tew et al., 2012), controlled trials using standardised measures of recovery would better determine the effectiveness of the supports provided by HC in facilitating personal recovery in participants. Further research is required to explore the relationship between fulfilling basic and psychosocial needs and the attainment of personal recovery among people with SMHCs. Integrating client-reported outcome and recovery measures will enhance future investigations and support more person-centred approaches to recovery. Peer support groups for people with SMHCs, particularly those in rural Australia, appear to make a major contribution to recovery among people with SMHCs. However, there continues to be a paucity of evidence on their role and usefulness. This study contributes to building the literature in this field. Considering the value that peer workers bring to the mental health workforce in rural areas, ways of growing the rural peer workforce are being explored (Nic Giolla Easpaig et al., 2024).

This study has some limitations. For instance, this was an evaluation of the work and outcomes of a single rural mental health peer group. It may not have captured the full diversity of experiences of people with SMHCs in rural settings. Obtaining participation of individuals with SMHCs in research is challenging and requires a lot of support from trusted individuals. Some interview questions may appear to be leading rather than open ended. All participants were currently engaged with HC and may have felt reluctant to share negative views about the group. Furthermore, the potential influence of other supports that participants may have received concurrently make it difficult to attribute outcomes solely to the role of HC.

## Supplementary Information

Below is the link to the electronic supplementary material.

Supplementary Material 1 (DOCX 36.0 KB)

## Data Availability

No datasets were generated or analysed during the current study.

## Declarations

Author AI’s salary is funded by the Australian Government Department of Health, Disability and Ageing, through the Rural Health Multidisciplinary Training [RHMT] program at Monash University. Author AB works at the Mental Health Services of Latrobe Regional Hospital, Traralgon.
