# Supplementary material for: Facilitating Personal Recovery in the Community: a Qualitative Evaluation of a Peer Support Group for Adults with Severe Mental Health Challenges in Rural Australia
Source: Community Ment Health J. 2026 Mar 12;62(6):1141–53. doi: 10.1007/s10597-026-01600-1 (PMC13451478; doi:10.1007/s10597-026-01600-1)
Supplement: Supplementary file 1 [file 10597_2026_1600_MOESM1_ESM.docx]

Supplementary file (Categories, codes and representative quotes)

# Difficulties faced by persons with MHCs

### Isolation

Due to community stigma

Like, when you get diagnosed with a mental illness, it comes like a shock as it normally would, you know. But it's not just about that. It's about like… you feel isolated from the rest of the community because you don't know anyone [who] has the same illness and when you talk to them about it, they look at you like you're crazy or you're gonna stab them or something like that, you know? – 1M

Due to fear of rejection

I went out, not on my own at all for that period of time. And, just that incredible fear of rejection and confrontation and all that kind of stuff. – 10F

Due to lack of support services

I mean, I've known of at least five people who have self-harmed or successfully committed suicide, because there's been nowhere else for them to go. They've felt that there's nothing left to do… there's nowhere to go… there's no support, and they've seen no way out but just leaving… and in my [case] I've had those same thoughts. … We are in a rural area, but we shouldn't feel so isolated in regards to support services. – 4F

Due to homelessness

Then the real estate [people] made it abundantly clear they're about to put the rent up from $215 a week to $375, and I could no longer afford to be there. So, I packed up and left in my ute [pickup truck] … and went to live in the bush [forest]. I had been in the bush for a while… and I realized after some soul searching that I was just incredibly lonely for human contact. – 13M

### Homelessness

There is a fair degree of homelessness or couch surfing. Well, it's very cold where we are. Like, this morning, I think it was six degrees. So, the winter weather is a huge problem for all of us and particularly if you're sleeping in your car or whatever. Some of them… they sleep in the shipping containers with nothing in them, you know. So that's a huge thing. – 10F

### Discrimination

If you have mental health problems in this town, you’re a second-class citizen… they treat you like garbage anyway, the hospital. – 3M

### Disrespect

But I know, as a person with mental health, I don't want clinical, I don't want white walls, I don't want someone dressed in a business suit to tell me how I live my life, you know? Don't write people off. – 16F

### Loss (of identity, self and relationships)

I, with the diagnosis and the unravelling of my life prior to that, I suddenly did not know who I was, anymore. Yeah. What do I like to do when my brain's actually functioning even on an everyday level? What do I wanna do? Like, I know, I can't sit at home forever. – 10F

I got a diagnosis, but, other than the clinical, perspective with the psychiatrist and psychologist, I didn't really know many people with a similar one, and it was hard to talk in general as friends with my other friends because they didn't quite understand. And I lost [friends] and that was hard just because of the stigma that came with the diagnosis. – 11F

### Worsening of mental illness

I was starting to get to the point where I was talking to myself in my bedroom and stuff every night, and I was I was going back into my delusionary state. I've been a gamer my whole life and … I view everything through this computer screen. – 1M

# Supports provided by Happy Chat

### Welcoming and non-judgemental

It's just a place that is you can catch up with people that are nice people and friendly and welcoming and have something to eat and drink…there's a community garden set up at the train station and it's in their shed that we meet. – 2F

… they said come along, and, you know, it's just simple, not too much fuss. You can come whenever you want. You don't have to stay long, you know, just whatever you're comfortable with. – 11F

I felt like I was welcomed, and I wasn't overly challenged as in people just let me be me … and so I found comfort there. That's really important because, my anxiety, takes the form of agoraphobia and so whenever I'm outside of my comfort zone, I just feel like I'm going to be ill. I feel very sick. -13M

So being new to the area, I find it very welcoming. I find it nonjudgmental…and it's okay to be you. People are okay to chat with you and accept you for who you are. – 2F

No judgement whatsoever, you know... So it is for people who are struggling, have no place to go normally, and they're looking for some place where they can, you know, really be themselves and share what they're going through and have somebody understand and listen to what they are talking about. – 16F

### Sharing a meal and a chat

You share a meal or some food and have a have a chat, have a happy chat. Yeah… It's nice to go up there and just sort of sit in the smoking area that they've got… this makeshift smoking area and just have a cigarette with others and just talk about video games and what's going on in people's lives and health conditions and all that sort of thing. – 1M

The meals! That's one of the biggest things that I reckon as a success at Happy Chat is that we have a meal together. Such a simple thing, but breaking bread together is a huge part of getting to know [each other]. So, access to food is amazing for your mental health… just to sit and eat together. Sometimes they take home leftovers. – 10F

### Inclusive

So, you know, there were weeks that you could turn up, and you just didn't feel like interacting with anyone, talking with anyone. But you still went because you enjoyed being there… and, like, you know, there were areas set up if you wanna be engaged in the group… but nonverbal. ‘We're happy for you to sit over here, if you wanna sit over there, we're just happy that you're here, and we will accommodate what you need for that hour and a half that you're here.’ – 4F

I was very shy. So at first, I was stuck in the corner, and there was always some very nice people who would come and talk to me even if I was being shy. And they tried to, you know, integrate me into the group, which did work. You know, that was very nice. – 17F

Oh, on a regular basis, probably between fifteen and twenty and some of them are vegetarian, some of them don't have capsicum, some don't have onion. So you've gotta be very careful with what you cook. And so, we we've just got it all down pat now. Yeah. If it's not gluten free, then they'll do a little gluten free menu for me. but some weeks, the food bank is not in plenty. – 4F

### Safe space

I think one of the most important things that stands out for me is the fact they had made that a very safe environment for all people. Different people have different issues. I think they had a sense of freedom. They could be themselves, and they weren't being judged by people. It was a lovely environment. – 12M

One of my sons has serious anxiety; So, safety is a huge issue for him. He feels safe at Happy Chat. – 15F

I know that I can go there if I'm feeling sad or happy or, you know, the different feelings… Either way, I'm always welcome and made comfortable. I still get anxious and things, but with Happy Chat, it really is like a group of friends. So, I feel just more emotionally comfortable having a place to be where I can talk about my feelings if I want to, that's safe… because everything's confidential. – 11F

They made me feel like people cared about me and they helped with my self-esteem quite a bit too, especially because people with similar struggles were able to relate to me. It was really helpful. – 17F

### Human Connections

Well, I talked to people, you know. I made some friends… instead of me feeding myself my own delusions, I was focusing on what was said at Happy Chat and the people that I met… and thinking about ways that I could help and do things with other people. And I think that was probably the most valuable thing that Happy Chat has provided me. It's the human aspect that's valuable to me. – 1M

Happy chat has just filled the void. I haven’t had anywhere else to go. I had separated from my family and had not spoken to them for about seven or eight years. Happy chat helped me with that. – 3M

I mean, we range from 10 people to 30 people sometimes. And … I know 95% of all those people's names. I see those people downtown. If it wasn't [for me] going to Happy chat, I wouldn't have been able to make those relationships… to be able to talk outside of Happy Chat. So, it's extended my network, if you wanna put it that way. – 5M

And suddenly I had a new group, a new tribe around me. – 10F

...you are able to meet a lot more people and, you know, be part of, some kind of group that you're comfortable in. – 8M

It is very much like a family. You sit down. You have a coffee. You have a meal. You know, it's in a broken down old shed that leaks from the roof when it rains. It's cold and drafty [sic]… Like, it's just very basic… -16 F

### Building hope through kindness

Sometimes, just a simple act of kindness or words from somebody else even if they didn't know what you're going through. So that hope will then carry me on for the rest of the day, and I won't feel utterly hopeless and in despair. Yeah. And that does make all the difference sometimes to whether I feel like I should be here or not… Yeah. – 14F

### Social and emotional support

Socialising and… being able to talk about difficulties that I’m having… Being able to talk to people and make friends and talk to [the coordinator]… talk out my difficulties and bring em [sic] out so that I could talk to other people about it so could tell me how to do things or help me with them. – 6M

I just sit down and talk to them. Initially I struggled to draw them out because, obviously, I was new there. So, I had to give [of] myself. So, each week, I talk to a different person. I talk to everyone in general, but I try to give at least fifteen minutes to a different person each session. And then draw them out asking about them and so forth. I quite enjoy that. So, yeah, it's been nice… I think to them, it shows that you're sincerely interested in them, and they open up, to more things. – 7F

Every time I talk about my problems… my problems are hard. So, just by voicing what I'm struggling with… lessens the load that I'm carrying. You know? it sort of just helps relieve that bit of tension and keep going. Keep moving forward one step at a time. -16F

You know, they've always been there for my mental health stuff, you know. When I had a really difficult time with some domestic violence, they actually all kind of banded together to help me with my pets and things. So they've been very helpful and very nice. And they've checked in on me since… - 17F

### General support

Happy Chat opened a door to a bunch of people who reminded me that I was not alone. In person or in my struggle with life. And it brought out my best. I have been financially supported by HC to make it all happen. When I was legitimately too poor to put fuel in my motorcycle to get there, I was offered gift cards for the servo [fuel station]. When I was really struggling, I was offered counselling that I didn't even know how to ask for, or believed that I was deserving of. Food, drinking water... [The organiser] dug into budgets that she worked hard to secure to truly 'support' me in every sense of the word. – 13M

Sometimes they might need help with paperwork or any sort of, support in whatever areas that [the organiser] might guide them or bring information for them to look at or share so that they know where to go for the help. – 7F

Happy chat helped me to get a council house. Don’t have to live in a caravan park any more…I have a friend who lives in a shitty old caravan. He comes here and he’s my friend… we talk a lot. I told him he needs to apply for council housing. – 18M

And they actually helped pay for therapy for me for a while until I got on the MBS[Government subsidised Medicare services]. – 17F

### Helping each other

We had access to some of the food bank. And, there was one time that there was period products and stuff like that as well, which was good. You know, one of the members bought me clothes one time. She didn't fit into them anymore. So, it's a lot of people helping each other. – 17F

So, I've helped one of our guys out because he doesn't have email. He doesn't have a clue how to use it. So, he was able to get some funds back there and he was just like, ‘I'm so grateful that you guys are here to help us with that… - 4M

### Enabling agency

It is also a good place aside from being part of the community, feeling empowered, and making choices… whether they want lunch today or whether they don't, would they like us to pack some up to take home or whatever. I know they're very simple but there's still choices. – 15F

### Monitoring wellbeing

We're like a monitoring facility. We can see how people are coping or not coping. We cannot single them out in the authoritarian sense, but just take them gently aside and ask how they're traveling. Are they okay? Is there anything perhaps we can do beyond happy chat? Either link them with… if they don't have any funding, perhaps [help] with that. So, it's a very holistic approach, It's simple. It's actually like family. – 15F

### Crisis care and support

I presented in an ambulance, suicidal thoughts… that's really bad…bad space… But luckily, I had Happy Chat. They were monitoring me, keeping an eye on me, which was really good. They'll just pop in [at home] and say, ‘Hey! How are you going? … Come for a drive! Yeah. Just different things. Just engaging me with different activities and stuff… - 4M

… they are not shut over Christmas. It’s always open. Even if you go at 2 o’clock at night into hospital, they are in there seven, eight o’clock the next morning, if they find out about it which I usually tell them. They are there to check to see if you’re alright. – 3M

### Advocacy

Carers Queensland would come down, every month…and she knew the people there; so she would organize meetings with them and the client. She'd be saying, ‘Look, if you need me to come and advocate for you, I'm happy to come and advocate for you’. Same with the hospital… anything that people said, ‘Look, I just need someone to be there with me’. and she's [the organiser] happy to do it. – 4M

### Continuing connections

There's a lot of people you won't hear from who have been Happy Chatters. Two main reasons being, some are just way too socially anxious. But others are success stories like myself. People who stabilized their life through difficult times at Happy Chat and went on to not need them anymore. These people are all around this district in a 50km radius from the middle of town to the forest along the border. And they all speak fondly of it… And they always ask after it and [the organiser] etc.. So even after people are long gone from it.. It still brings people together. – 13M

### Continuing support

We need to be able to go somewhere safe to be able to meet people… and be safe and get that support if you need that. You know? And you don't need that support for six weeks in a row. You need that support, theoretically over the year or over the span of [your illness]. So, you might be good for two months, but then you might have a really bad time for a month, and you need that extra support. And that's what Happy Chat offered. – 5M

I think its greatest value is that it's a constant. It's a constant in people's lives; that every Tuesday morning, there's something to get out of bed for. – 15F

# Outcomes achieved

### Motivation to leave the house

I was newly diagnosed, and extremely isolated, and, I didn't get out of the house at all; and, my husband knew about Happy Chat, and he suggested it to me. And, he came with me, which was a big deal to go the first time. He came with me, and then after a while, I was coming on my own. So, it enabled me to get out of the house. – 10F

My client doesn't like to leave the house very often… he has social fears and phobias I guess, and definitely doesn't like crowds... So, it is a great way and a great encouragement for him to get out of the house. – 2F

### Improved confidence

… he's comfortable going into a public space. And yeah, it might only be for half an hour to have some lunch or something, but he's still going out. So [Happy Chat is] a safe place to start and is accepting, and that sort of builds up their confidence, and then they can do it elsewhere. – 2F

I've [gone from being] lost to building my confidence level back up again. I've lost 18 kilos of body weight because of that self-confidence. The ability to go out and do it without being too afraid. And I do suffer high anxiety, so I'm always looking over my shoulders. I'm always doing that. I didn't wanna leave the house because of anxiety, but I've now got that. – 5M

… this person was coming along with his support worker. And initially, [during the] first few weeks, he just would sit there and then go; wouldn't eat; wouldn't talk much… But as the months progressed, I have seen him participate in the garden work as well as enjoying the meal and actually smiling and talking… So it also helps them by building confidence bit by bit; they can go through this … they're not alone dealing with their issues. – 7F

My dad helped me to get to a few of those Happy Chat sessions, and then eventually, as I felt a bit better, I'd be off by myself. I didn't have to have him there. – 11F

Yeah. Happy Chat was the first thing other than doctor's appointments. After probably three or four weeks of Happy Chat, I was then able to go on my own. That was the only thing I went out to every week. Well, I actually found it really hard to talk at first because it had been so long… - 10F

You're not afraid to talk about stuff and suggest things and you can help other people with your suggestions and they can help you. You know, Joe Blow down the road… He's got a room for rent. You could try there or whatever. He's ended up renting a room from another person who goes to Happy Chat. – 2F

I actually can go shopping on my own now. I’ve actually started going to church without feeling paranoid. I’ve become a little bit more open. I can go into a café I can talk to the staff … yeah, its made a huge difference like that.. I’ve become more confident. Like I’ve started going to the Gym three times a week and joining classes talking to people there… I’m getting fit. I’ve got a lot of confidence to do things. – 19M

### Enabled a sense of belonging

Once I got over my initial shyness and nervousness, I could feel like I could be myself. and that was really helpful. And I still get social anxiety… So, you know, sometimes, and depending on the day... but, yeah, it's helped me feel like I belong, yeah. – 14F

There is nothing nicer than seeing someone familiar in the main street and saying “hello”. The smile and the hug back is life giving. The sense of belonging to our community and being an active participant in it, is priceless. It brings a sense of safety but also an opportunity to find joy in belonging to a community. – 9F

### A place of comfort

I felt comfortable. I managed to make this venue that they were using for Happy Chat one of my comfort zones. And so, I could feel absolutely lousy on the way there, but once I got there, I actually felt comfortable. And that was probably the biggest breakthrough for me because if I haven't been able to get comfortable there, I wouldn't have been able to navigate the social challenges that came next. – 13M

So after probably three or four weeks, I was able to go on my own without my husband because I felt comfortable there. I knew where to walk in, where to get my cup of coffee. And by then, I was feeling more comfortable talking again, and was getting to know [the coordinator] a bit better too. – 10F

### Safe to be me

And I've noticed, different changes in different people where they're much more open now. And I presume they feel like they are accepted. I suppose even then I'd have to consider myself, probably being rather guarded, being careful when I first started off. Now I can be myself. I don't have to be anybody else. I can be me, and they all accept that. – 12M

### Improved health and wellbeing

Happy Chat got me out of my skin and out of my house, out to work, outside. I still suffer from depression, anxiety, all that. I mean, I go into supermarkets, and I'm still highly agitated… but at least I can walk, health wise. I've got healthy. I'm losing weight. I've got a, a companion even though it's early days, but I got a companion. And that was all through Happy Chat. – 5M

### Improved self-care

I’ve actually started looking after myself, self-care and things like that…wearing clean clothes and having a shower every day and brushing my teeth and have more control and I’ve lost weight…The self care side of things made my wife very happy. I mean I am a little bit untidy and that sort of thing but I am more helpful around the house. I make sure things are clean and tidy and put away when I can. – 19M

### Improved social life and social skills

I got more social skills… I didn't have many. I barely left the house. I was quite reclusive, I would say. You know? It gave me something to look forward to. And so that was something that was a big impact for me. I'd only leave the house once a week to go to Happy Chat… -17 F

I’ve got more friends… sort of feels like a family because my brother's three hours away. Yeah and the community family up here really makes me feel welcome. – 6M

I am mostly socially awkward. I don’t mix with other people. A lot of that has to do with my autism. I’m not a social person but since I’ve been going there, I’ve become more social. I’ve got to talk to other people and drop my guard a bit there too. I’ve got trust issues too but I’ve learned to be more relaxed, be more myself around these people, which has definitely made a huge difference. – 19M

### A new identity

Over the years that I was at Happy Chat, I tried different things, but did manage to redefine or find a new identity or whatever or maybe my real identity in the long run anyway. What I was about, what my values were. It was a lot of… being able to rediscover who I was, who I wanted to be, what my values were, and things like that. So, it was like, … If it hadn't been for them, I don't know what I'd be doing now. – 10F

### A new sense of purpose

And also, when we did the art as that separate program with the Happy Chat people, we were making things for the community, which sort of gave us, like a sense of purpose. It made us feel good to help make the community better. We made these sculptures that we put in the community garden. -11F

After psychosis, I lost track of who I was and what I wanted to do with my life. Attending then running HC has given my life meaning and helped me to focus on my personal and citizenship recovery. Also in my role coordinating HC, I have discovered that helping others feels good, which in turn, keeps me motivated to keep going. – 9F

### A new sense of hope

We first come to Happy Chat with the idea that all we are is a mental illness; that we are sick and disabled… When people discover that they are not defined by their mental illness, that their lives are richer than a diagnosis… there is enormous relief for the individual. More than that, it reminds them that they aren’t hopeless cases - that they are worthy of a good life… Many had lost hope before HC - I know I had. Not anymore! – 9F

### Employment

I've seen [others] come to Happy Chat even for help with getting community help… you can see their journey, you know, they might have started being depressed in the first month or two, but then in the few months after, maybe they're a bit happier… there was one person that even managed to get stable that they could get a job… all of a sudden!! They had been unwell for a long time before that. – 11F

### Realisation that it is the system that has failed and not us

Before Happy Chat, I believed I was alone in my experiences. I believed I was broken. I was the reason I wasn’t getting better and that I should be grateful to receive any assistance. I felt the same for my partner. It was insisted that we were broken, that we were the failure, we weren’t working hard enough. But it’s the system that is indecently flawed. HC helped me realise that we all had the same shared experiences. Diagnoses aside, our treatment was uniformly traumatic and distressing. Sharing our stories also helped highlight just how much further we needed to go in formal mental health supports in community, and perhaps why existing supports are failing us everyday. – 9F

# Continuing challenges

### Dependence on one person

That's what I'm worried about… [The organiser] because she is under the pump. She has a lot on her plate for the community. And that's the only concern because if she breaks, it all breaks. – 16F

It would be good to have more people stepping up to take the reins of Happy Chat. Like, at the moment, [the organiser] and I pretty much do everything and that can be very wearing after many years. – 8M

### Lack of funds

Happy Chat doesn't have any money… We've had donations in the past to pay for food, and she gets food from the food bank or Second Life or whatever it is called. – 2F

Some weeks, we cannot take anything. So, we're then left to look at what kind of funds do we have left? I do the ‘cash for cans’. So any cans or bottles that I can get, I collect from the wineries around here and my neighbours, they've all got those bags. So they just dump them in the bags and bring them over, and we cash them in. – 4F

As far as I know, there's a very, very tiny budget. It might only be 20 or 30 dollars a week to buy the milk and the tea and coffee and all that kind of stuff, and then some stuff they grow in the garden, they use. So that's not adequate, you know. Like, there's a lot of very low socioeconomic, situations for most people there. – 10F

### Managing difficult behaviours

Some became aggressive and they were told that they would have to be banned from coming for two weeks and I think one person in particular… unfortunately, went too far and they were told, ‘Please don't come back.’ you've got a hard decision to be made Yeah… But you've got to think of the welfare of the greater majority– 12M

### Continuing discrimination in society

People are always judging, aren't they? whether it's for their safety or whether they wanna be your friend or… you know, so that's still a difficult thing because … it's our societal conditions and constructs. I guess that we live at the time we're in right now. And I guess over years and so on, that would change and hopefully evolve for the better. – 14F
